# Lessons from ten years of crystallization experiments at the SGC

**DOI:** 10.1107/S2059798315024687

**Published:** 2016-01-22

**Authors:** Jia Tsing Ng, Carien Dekker, Paul Reardon, Frank von Delft

**Affiliations:** a Structural Genomics Consortium, University of Oxford, Roosevelt Drive, Oxford OX3 7DQ, England; b Center for Proteomic Chemistry, Novartis Institutes for Biomedical Research, CH-4002 Basel, Switzerland; c Swissci AG, Industriestrasse 3, CH-6345 Neuheim, Switzerland; d Diamond Light Source Ltd, Harwell Science and Innovation Campus, Didcot OX11 0QX, England; e Department of Biochemistry, University of Johannesburg, Aukland Park, Johannesburg 2006, South Africa

**Keywords:** crystallization screening strategy, sparse-matrix screens, large-scale data sets, data mining, redundancy of conditions

## Abstract

Although protein crystallization is generally considered more art than science and remains significantly trial-and-error, large-scale data sets hold the promise of providing general learning. Observations are presented here from retrospective analyses of the strategies actively deployed for the extensive crystallization experiments at the Oxford site of the Structural Genomics Consortium (SGC), where comprehensive annotations by SGC scientists were recorded on a customized database infrastructure. The results point to the importance of using redundancy in crystallizing conditions, specifically by varying the mixing ratios of protein sample and precipitant, as well as incubation temperatures. No meaningful difference in performance could be identified between the four most widely used sparse-matrix screens, judged by the yield of crystals leading to deposited structures; this suggests that in general any comparison of screens will be meaningless without extensive cross-testing. Where protein sample is limiting, exploring more conditions has a higher likelihood of being informative by yielding hits than does redundancy of either mixing ratio or temperature. Finally, on the logistical question of how long experiments should be stored, 98% of all crystals that led to deposited structures appeared within 30 days. Overall, these analyses serve as practical guidelines for the design of initial screening experiments for new crystallization targets.

## Abstract 2

Observations are presented from retrospective analyses of the crystallization strategies deployed at the SGC, Oxford during its first decade of existence, providing practical guidelines for the design of screening experiments.

## 1. Introduction   

One of the promises offered by large-scale structural genomics efforts, which first launched at the turn of the century, was that the extensive accumulation of data would help to demystify aspects of crystallography that were limited to, or certainly dominated by, anecdotes and the confirmation bias of experienced practitioners. Crystallization was a particularly notorious process, and many studies did emerge from the large-scale facilities with real practical consequences for the ever-growing protein crystallography community (Asada *et al.*, 2013 ▸; Burley *et al.*, 2008 ▸; Elsliger *et al.*, 2010 ▸; Rupp *et al.*, 2002 ▸). Rigorous verdicts on the various strategies remain unavailable, however, as they are difficult to arrive at objectively, even though these are most relevant to the day-to-day crystallographer in pursuit of a biological rather than a methodological question.

The Structural Genomics Consortium (SGC), Oxford deposited its first structure in the Protein Data Bank (PDB) on 20 April 2004 (PDB entry 1t2a). At the cutoff point for the current analysis, 31 March 2014, SGC Oxford has contributed 700 deposited X-ray structures, 462 of which were novel at their time of release (*i.e.* the first representative X-ray structure of that polypeptide or a particular protein–protein complex, within 95% homology). The methods and practices implemented over the years for solving these structures have been outlined in past SGC publications, including the strategy for cloning, expression and purification methods used in the laboratory (Savitsky *et al.*, 2010 ▸) and the techniques and procedures for data collection at synchrotron sources (Krojer *et al.*, 2013 ▸). This work bridges these publications through retrospective analyses of the nano-droplet crystallization strategies employed at the SGC.

As in many crystallography laboratories, robotics and automation are crucial to our crystallization facility. Emphasis was placed on making it accessible to any protein scientist to ensure that only protein quality and quantity should ever be limiting, rather than the expertise or non-protein materials required to carry out the crystallization experiments. Standard crystallization practice was implemented largely by ensuring the recommended experiment was also the most convenient, including ensuring that primary sparse-matrix screens and plate types were always adequately stocked, predefining liquid-handling protocols with obvious names and setting up relevant defaults in the software and databases.

As a result of this philosophy, more than 200 scientists used the facility over the ten-year period covered here, generating over 60 000 crystallization plates from 2339 targets and over 24 000 protein purifications. Of all targets for which purified protein was ever available, 43% eventually yielded diffracting crystals.

The focus of this paper is the first phase of the crystallization process, namely the coarse screen, *i.e.* the screening of conditions for crystallization (McPherson & Gavira, 2014 ▸). Shaw Stewart & Mueller-Dieckmann (2014 ▸) highlight that there are four main parameters for initial screening experiments: (i) the ratio of sample to crystallization cocktail, (ii) the incubation temperature, (iii) the number and choice of initial screens and (iv) the crystallization method. Our data and analyses inform the first three, but provide no insight into the fourth, because sitting-drop vapour diffusion was the only format compatible with high throughput in our particular hardware configuration.

We reviewed different aspects of the crystallization strategies employed and report the conclusions from these, focusing on (i) repeating screening conditions with different protein-to-precipitant mixing ratios; (ii) repeating screening conditions at different incubation temperatures (277 and 293 K); (iii) the choice of sparse-matrix screens; (iv) the protein sample concentration for crystallization; and (v) the storage time of crystallization plates.

Given the retrospective nature of the analysis, all observations pertain to the strategies actually used to accumulate these data and say little about orthogonal approaches. Nevertheless, since our procedures were designed taking into account actual costs, the conclusions drawn should be helpful whenever setting up crystallization screening experiments.

## 2. Data set   

**Figure 1:**
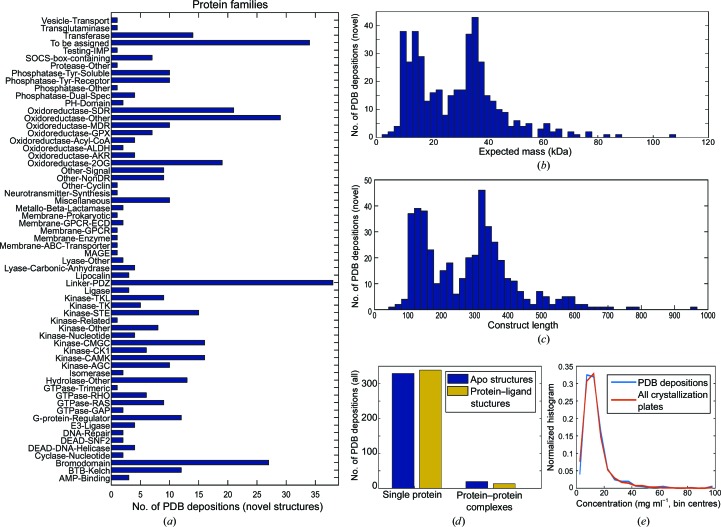
Summary of novel structures deposited by the SGC, Oxford over ten years. (*a*) 61 protein families are represented, as annotated *a priori* based on bioinformatics. (*b*, *c*) Masses range between 2.5 and 108 kDa, with chains of up to 967 amino acids in length. (*d*) The majority of structures were single protein structures, either apo or ligand-bound; a small proportion were protein–protein complexes. (*e*) Comparison of protein concentrations in crystallization experiments for those that led to PDB depositions (blue) and all crystallization plates (red). Both populations have a very similar distribution.

The SGC has focused on human proteins from multiple families. Fig. 1 ▸(*a*) shows the families of deposited novel structures over the period analysed, along with the distribution of mass (Fig. 1 ▸ *b*) and protein length (Fig. 1 ▸ *c*). Protein classes not explored at the SGC, and hence not informed by this analysis, include viruses and large complexes. A few protein–DNA and protein–RNA complexes are among the structures solved in this period, as are a very small number of integral membrane proteins. However, these classes are not represented in our analysis owing to their negligible representation. The structures deposited comprise of apo structures, protein–ligand structures and protein–protein complexes (Fig. 1 ▸ *d*).

Preparation of protein samples was performed by almost 200 scientists, who varied purification protocols at their own discretion as deemed necessary depending on sample. However, the dominant strategy was affinity chromatography, primarily by His tag/nickel but also glutathione *S*-transferase tag/glutathione, followed by size-exclusion chromatography; a final ion-exchange chromatography step was occasionally used. Millipore centrifugal parallel membrane concentrators were the standard device for concentrating proteins; as the primary constraint was protein stability and the goal was to avoid precipitation of protein, a concentration of 10 mg ml^−1^ was the target in the first instance. The routine workflow did not include a systematic protein concentration test, and attempts to introduce it fell on barren ground, presumably because it does not provide a clear either/or read-out, and with the infrastructure in place it was evidently easier to set up the crystallization experiment. The distribution of concentrations of proteins for crystallization is shown in Fig. 1 ▸(*e*): the majority of experiments, including those that led to PDB depositions, were concentrated to no higher than 20 mg ml^−1^.

In line with common practice in laboratories with robotics, the crystallization strategy was to set up multiple sparse-matrix (coarse) screens (Jancarik & Kim, 1991 ▸); the screens used in meaningful quantities are summarized in Table 1 ▸ and three of these were in-house designs.

(i) The Ligand Friendly Screen (LFS) is based on the PACT screen (Newman *et al.*, 2005 ▸). It samples combinations of PEG 1K to 6K, salts and buffers at near-physiological pH in a semi-systematic way: the goal was to obtain crystals that were directly usable for studying compound binding.
(ii) Basic ChemSpace (BCS) consolidates a wide diversity of PEG precipitants into a single screen by using four PEG mixtures or ‘smears’ grouped by molecular weight, thereby retaining the potential specific properties of a given polymer size without a combinatorial explosion (Chaikuad *et al.*, 2015 ▸).
(iii) JCSG+ is similar to the commercial version (Newman *et al.*, 2005 ▸), but was optimized with the author’s help to reduce the number of stock solutions required.

All screens were mixed in-house with a MultiPROBE II Plus HT/EX Robotic Liquid Handling System (Perkin Elmer; http://www.perkinelmer.co.uk) until 2013, and since then have been commercially sourced from Molecular Dimensions (http://www.moleculardimensions.com), including the in-house designed screens. Over the period analysed, scientists could request or design optimization or fine-grid screens themselves, with a laboratory technician available to formulate the requested screens robotically using commercial stock solutions. The goal was to make it as simple as possible for experimenters to optimize hits from coarse screens if the original crystals did not satisfactorily diffract sufficiently.

The standard crystallization screening experiment entailed two to four sparse-matrix screens with three 150 nl droplets per well prepared by mixing protein and precipitant in ratios of 2:1, 1:1 and 1:2. Prior to February 2009, the primary plate type used was the CrystalQuick Plus 96 Well (Greiner; flat bottom, three subwells, available from http://www.hamptonresearch.com), with 80 µl reservoir solution; after that, 3 Lens Crystallization Microplates (http://www.swissci.com) were exclusively used. This plate type has three concave subwells per well and was developed by Swissci in partnership with the SGC by modifying the MRC 2-Well Crystallization Plate to accommodate our preference for three droplets per condition, and to additionally lower the reservoir volume to only 20 µl leading to lower reagent consumption.

Crystallization droplets were mixed with Mosquito Crystal robots (http://www.ttplabtech.com) robots at room temperature, and plates were incubated at 277 or 293 K, with drops automatically imaged at fixed time intervals by Minstrel HT systems (http://www.rigaku.com). The inspection schedule was nominally 1, 2, 4, 7, 14, 28 and 56 days from setup, with the time tolerance being tighter for early inspections; the actual inspection times varied with imaging load and backlogs. Vendor software was set up to enable experimenters to view and score their own experiments from their desk. All crystallization experiment information, including details of the protein, its preparation and its co-crystallization compounds, along with downstream experiments up to deposition in the PDB, were recorded in an in-house database; user-assigned crystal drop scores were also copied to this database from the vendor’s imaging database.

**Figure 2:**
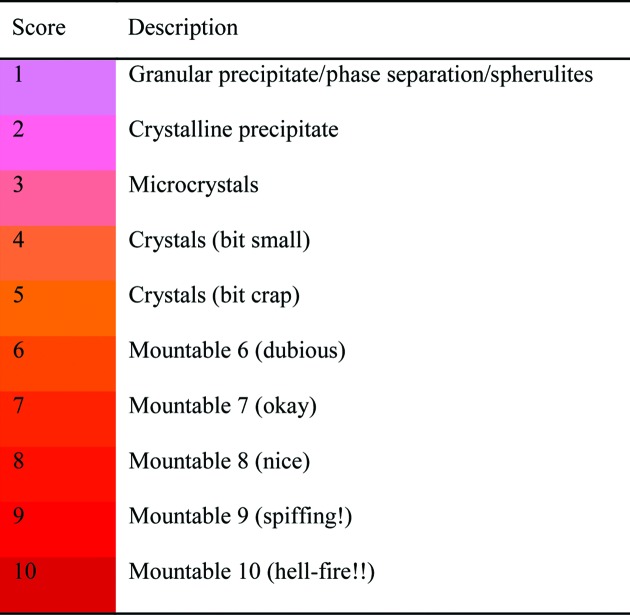
The scoring system used at the SGC for recording crystalline behaviour, Scores of ≥3 are considered to be hits. Other scores/classes for precipitates and droplet errors are not shown. The tags and colour scheme are reproduced here exactly as they are configured in the software, since this is the only description of these categories that was ever seen by all users (although most were given training by more experienced scientists). This sparseness of information was deliberate, since it was always assumed that scoring would be largely subjective.

Viewing images was optional, and typically only a few inspections were actually viewed, although viewing generally entailed seeing every drop of the inspection. Considerable care was taken in advance to achieve high reliability of scoring: the mundane problem of ensuring that the viewing client was always available and responsive required constant maintenance, while the database was configured such that recording a score the proper way greatly simplified the registration of downstream experiments. Finally, a scoring scheme was designed that would be both robust and intuitive (Fig. 2 ▸): to factor in the inevitable category errors (Buchala & Wilson, 2008 ▸), it was set up as a 1-to-10 sliding scale and scores were attached to vivid colouring of increasing intensity alongside descriptive yet deliberately subjective labels.

Although compliance is notoriously hard to assess, we feel confident in assuming that *most* instances of crystallinity were labelled, given that most of the many images per plate (up to eight inspections × 288 droplets = 2304) were indeed never labelled. In a previous analysis (Ng *et al.*, 2014 ▸) we concluded subjectively that the score of crystallinity is broadly reliable, although it inevitably carries significant error bars owing to its subjectivity. The sliding scale of Image Score was designed specifically to mitigate the effect of such subjectivity.

Crystals that were tested for diffraction with our in-house rotating-anode generator or at synchrotron sources were tagged with a Crystal Quality Score, which captures very reliably whether or not a crystal diffracted (any Crystal Quality Score ≥ 1 indicates diffraction, with higher diffraction quality receiving a higher score).

For the purpose of this analysis, we define the following:

**Hits** have an Image Score of ≥3, where the image was evaluated by an experimenter to contain microcrystals or larger, and there is at least potential for further optimization.
**Diffracting crystals** have a Crystal Quality Score of ≥1, where the crystal was subjected to an X-ray beam and produced protein diffraction patterns.

## 3. Protein:precipitant mixing ratio   

While sparse-matrix screens ideally yield well diffracting crystals directly, more generally they are also effective even if they generate only crystallinity (‘hits’), as this provides a quick read-out of whether the particular protein preparation is of sufficient quality. These also inform subsequent follow-up strategies. We analysed whether the practice of multiple mixing ratios in sparse-matrix screening experiments increased hit identification, *i.e.* whether the three-droplet strategy resulted in hits that would otherwise not have been found if fewer droplets had been employed.

To this end, we generated the distribution of crystallinity in all sparse-screen subwells. All human recorded scores for all 19 817 sparse-matrix plates set up since June 2004 and containing at least one annotated hit, were retrieved from our database. We considered all scores indicating crystallinity (3–10) to be hits, and the last recorded score was taken as conclusive. For each plate, the number of wells or conditions that gave hits in any subwell was identified: plates with no more than five crystallizing conditions were classed as ‘difficult’ experiments and the protein as a rare crystallizer, since they only crystallized in a narrow range of conditions; plates with more were classed as ‘easy’ and the protein was considered promiscuous. For each crystallizing well, the subwell(s) that gave the hit(s) was identified. All plates were treated as independent experiments, even though some would have shared the same protein: this analysis was meant to reflect a hit-identification exercise, which is the simplest form of analysing crystallization screening results.

**Figure 3:**
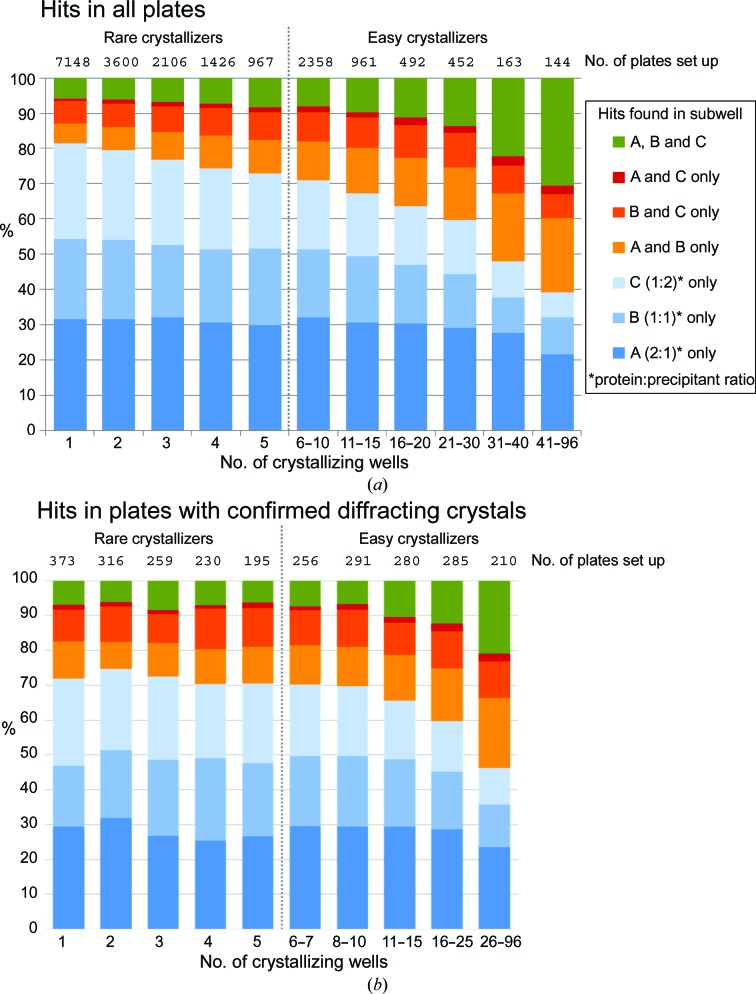
The use of multiple drops increases the likelihood of hit identification for rare crystallizers. (*a*) The frequency of finding hits in any subwell or combination of subwells was plotted for all crystallization plates of various levels of promiscuity, *i.e.* the number of crystallizing wells. For rare crystallizers (number of crystallizing wells ≤ 5) most hits were found in only one of the three subwells (blue shades). The number of wells with hits in multiple subwells (green or orange) increases if the protein also crystallizes in many conditions. Hence, omitting any one subwell would result in up to 30% of hits being missed. The total number of plates for each bar is given at the top of the graph. (*b*) The frequency of hits in subwells in plates with confirmed and tested diffracting crystals follows the same trend as in (*a*); the numerical variations are attributable to the necessarily smaller sample size.

The analysis is summarized in Fig. 3 ▸(*a*), and three observations support its accuracy. Firstly, hits occur in multiple subwells more frequently when there are more crystallizing wells (right side of the plot), which matches the intuitive assumption that if a protein is promiscuous and therefore crystallization is less sensitive to chemical conditions, the starting protein concentration will also matter less. Secondly, there is a low incidence of hits in category ‘A and C only’, which is an outlier category, because if both A and C are in the right concentration range to yield crystallinity, then B ought to be too. These outliers may arise from incorrect scoring, faulty droplets or stochastic nucleation. Thirdly, the overall trends are similar whether all hits are considered (Fig. 3 ▸ *a*) or whether experiments are selected more stringently, namely only those that produced crystals with experimentally confirmed diffraction (Fig. 3 ▸ *b*): this implies that visual scoring does broadly reflect true crystallinity.

The analysis reveals two important points. Firstly, for rare crystallizers (≤5 crystallizing wells, left side of the plot, 77% of the plates) each single subwell accounts for ∼30% of the hits identified: omitting any subwell would reduce the likelihood of identifying hits by almost a third for precisely those targets where one could least afford this. Secondly, the protein samples tended to be more dilute than what was appropriate for these sparse-matrix screens, since for all classes, subwell A (2:1 protein:precipitant ratio, which most concentrates the protein) produced more hits than did subwells B and C. This may well reflect how proteins have become more challenging, with generally achievable concentrations for human or eukaryotic targets being lower than for the typical non­recombinant target a few decades ago when the first screens were formulated. Concentrations in the original screens were attuned to the typical protein concentrations achievable at the time, where drop ratios were commonly 1:1, and screen formulations have been very resistant to change even as they have been assimilated into new screens.

Overall, we conclude that one cannot assume that the protein concentration one has achieved is ideal for a 1:1 experiment: exploration is required, and this is most easily achieved by varying the protein:precipitant mixing ratio. Indeed, closer inspection showed that for a given experiment it is not the same subwell that produces crystals in all wells with hits: even within a screen, exploration of protein concentration is productive.

**Figure 4:**
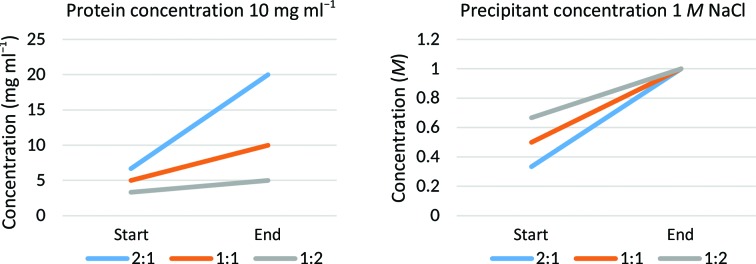
Both protein and precipitant concentration increase very differently for different mixing ratios. Plotted here are the calculated concentrations at the start and the end of a vapour-diffusion experiment in which a hypothetical protein at 10 mg ml^−1^ is mixed with an example precipitant (sodium chloride) at 1 *M* at mixing ratios of 2:1, 1:1 and 1:2. These numbers capture the case that the protein does not change phase and that equilibration runs to completion, even though the rate of the process varies considerably depending on the chemical components (Luft & DeTitta, 1995 ▸).

This complements other reports of the importance of replication for increasing crystallization success (Newman *et al.*, 2007 ▸), but our strategy of multiple mixing ratios additionally provides three sampling points on the phase diagram. The variation is likely to be considerable, as illustrated by the calculations in Fig. 4 ▸: the 2:1, 1:1 and 1:2 mixing ratios result in both the protein and the precipitant concentration increasing threefold, twofold and 1.5-fold, respectively, from initial mixing to equilibration, assuming complete equilibration with only the transfer of water vapour. Moreover, this introduces variations in kinetics beyond simple concentration effects: the 1:2 protein:precipitant ratio should have the lowest equilibration rate since the initial setup is closest to the reservoir condition; minor changes in salt concentrations in PEG-mediated vapour diffusion have also been shown to result in major effects in equilibration rate (Luft & DeTitta, 1995 ▸); and the three droplets may end up in different sizes and volumes, thereby affecting the kinetics of crystallization (Forsythe *et al.*, 2002 ▸). Of more direct practical use is that trends across the concentration gradient are immediately observable by comparing drops: especially for noncrystallizing wells this provides additional information on the interaction of the protein and the crystallization cocktail, as well as an estimate of the required increase or decrease in concentration.

## 4. Incubation temperature   

**Figure 5:**
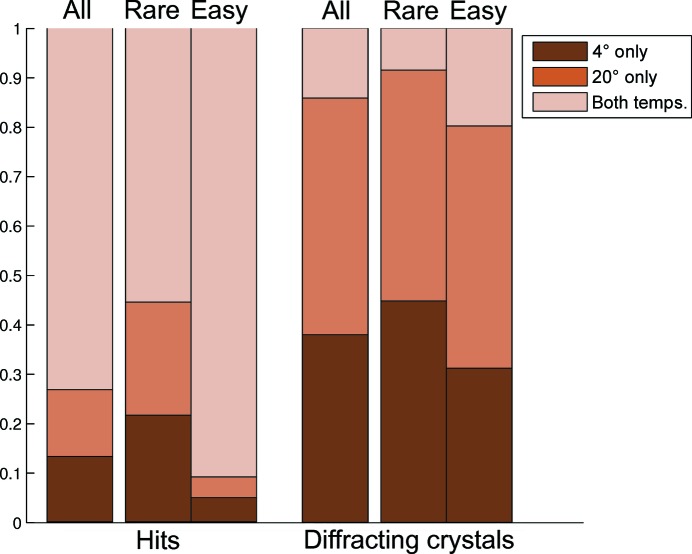
Percentage of experiments that crystallized only at 277 K, only at 293 K or at both temperatures for a total of 587 pairs of sparse-matrix screens. Rare crystallizers are those that exhibit crystalline behaviour in five or fewer conditions. Although the majority of hits were found at both temperatures, up to 45% of hits were identified at only one temperature for rare crystallizers. Experimenters were even more selective when testing the crystals for diffraction, mostly picking only one temperature.

We analysed the outcome of pairs of experiments set up at both 277 and 293 K to identify whether one incubation temperature was more effective at producing hits. In order to compare only the effect of temperature, we compared only pairs of experiments that contained identical sparse-matrix screens and the same protein sample (from the same purification batch, both either fresh or previously frozen), were set up on the same day with identical concentration for crystallization and compounds for co-crystallization (if any), and were incubated at both 277 and 293 K. We further limited our analysis to 587 pairs with associated diffracting crystals where at least one diffracting protein crystal was produced in either one or both plates of each pair. For each pair, we identified whether at least one hit was found at only 277 K, at only 293 K or at both temperatures, and likewise for diffracting crystals (Fig. 5 ▸).

Considering the incidence of hits (Fig. 5 ▸, left), we conclude that while promiscuous crystallizers are predictably insensitive to temperature, the use of multiple incubation temperatures was particularly important for rare crystallizers: 45% of hits appeared at only one or the other temperature and were distributed evenly between them, and omitting any one temperature risks missing >20% of hits.

Although this effect is apparently far more marked when considering diffracting crystals (Fig. 5 ▸, right), on further examination it transpired that this is an artefact of the details of how harvesting is planned and performed at the SGC. Crystals are typically pre-selected at the desk by examining the images only, whereupon the plates with the most attractive crystals are retrieved from the incubators and transported to the harvest rooms, several floors away. This is inconvenient to perform for more than one temperature at a time, since crystals are harvested at the temperature they are grown at; thus, if the first crystals to be harvested diffract well there is no incentive to harvest from other temperatures.

## 5. Sparse-matrix screens   

**Figure 6:**
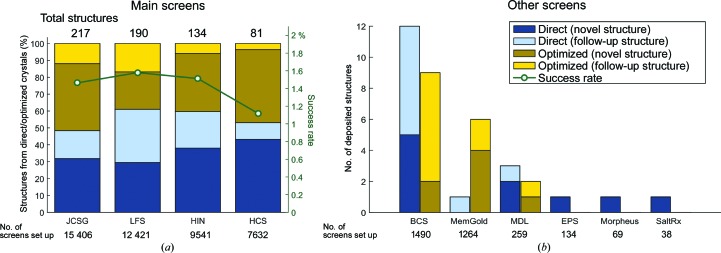
Comparable success rates observed for the four most popular screens used at the SGC. (*a*) shows the number and proportion of structures solved with crystals directly harvested from sparse-matrix screens (blue) or those that required optimization (yellow) for these screens. Novel structures and follow-up structures have also been separated as they do not necessarily have the same crystallizing conditions and hence are often re-screened. The green line indicates the success rate (percentage of structures derived from the screen/total number of screens set up). (*b*) shows a breakdown of the remaining deposited structures (6%) from other screens. See Table 1 ▸ for the abbreviations used.

We analysed the use of various sparse-matrix screens and the associated successes by identifying the screens responsible for all PDB depositions from SGC Oxford. A breakdown of the screens used and the associated success rates are shown in Fig. 6 ▸. Of the 700 structures deposited, 52% were solved with crystals harvested directly from a sparse-matrix screen, while the rest required further optimization, and for most of these (41% of total) the screen that yielded the original hit could be reliably identified from the database. The JCSG, LFS, HCS and HIN screens (see Table 1 ▸ for abbreviations) make up 93% of sparse-matrix screen experiments at the SGC, presumably because they were most reliably stocked, since no protocols were enforced as to how they should be prioritized. While our yield of structures direct from the coarse screen is high compared with other analyses (17–25%; Fazio *et al.*, 2014 ▸), we tentatively attribute this to the stringent enforcement of best practice for crystal testing and data collection (Krojer *et al.*, 2013 ▸). Specifically, this yielded usable data sets for many crystals that might have appeared unusable to less experienced practitioners.

These four screens were also responsible for over 94% of all deposited structures when counting crystals grown both directly in, and through optimization of conditions present in, these screens. A breakdown of these numbers is given in Fig. 6 ▸(*a*): success rates (green line) were calculated as the number of structures attributable to that screen divided by the total number of screens set up. Also shown are the proportions of structures solved from crystals directly obtained from the sparse-matrix screen (blue) or an optimized condition from the screen (yellow).

On the face of it, the older, original screen (HCS) was outperformed by the more modern screens (JCSG, HIN and LFS). HIN was explicitly formulated to cover regions of chemical space not covered by HCS, and JCSG was the consolidation of successful conditions from at least six commercial screens tested against hundreds of proteins. However, the fact that LFS has both the highest success rate and the most structures directly from coarse screens is particularly interesting: although its coverage of chemical space is narrow, by design it has high internal redundancy. This lends further support to the importance of redundancy, as discussed in the previous sections. On the other hand, this is a drawback when protein supply is limited, as discussed later, and the other screens sample chemical space significantly more widely than LFS (Table 2 ▸), which should be more informative as long as an efficient infrastructure is in place to design and mix optimization screens and thus pursue marginal hits rapidly. Such solutions are available from vendors (although expensive), but not at the SGC, where the home-grown infrastructure was initially rudimentary and later merely adequate; therefore, fewer marginal hits from the non-LFS screens will have been pursued, particularly whenever a ‘better’ hit had been identified elsewhere. Thus, the superiority of the LFS screen is misleading and is unlikely to reflect the true usefulness of exploring chemical space in the coarse screen.

**Figure 7:**
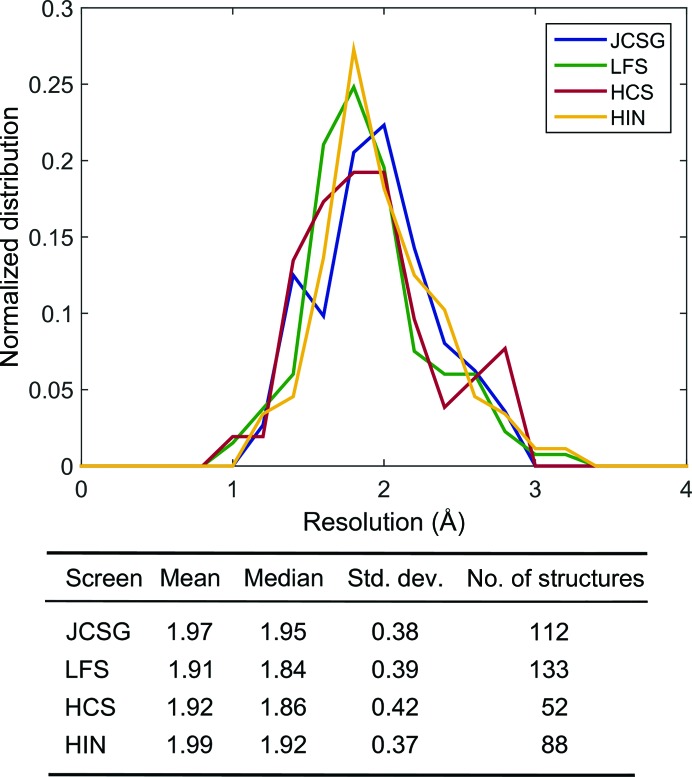
Relatively similar distributions of diffraction resolutions were observed for structures directly obtained from crystals from sparse-matrix screens with comparable statistical summaries.

The distributions of diffraction resolutions are similar for all four major sparse-matrix screens (Fig. 7 ▸); this is consistent with the assumption that it is the protein itself whose properties dictate crystal quality.

Other screens that also contributed to PDB depositions are shown in Fig. 6 ▸(*b*), although without showing success rates, as the number of samples is too low for these to be meaningful. The fact that these screens were so rarely used, even though they were always available, leads us to conclude cautiously that it is adequately informative to set up only a few screens (four in our case); the main caveat to this conclusion is that training bias is likely to be strong, since most users were not experienced crystallographers.

## 6. Recommendation for typical volumes of protein sample   

Combining this observation with our preceding conclusions, we can formulate a recommendation for what is a good volume of purified, concentrated protein to aim for when working with reasonably well behaved targets (decent expression and solubility): four screens each at two temperatures, with each condition in three drop ratios in 150 nl drops, consuming 172 µl of protein if dispensed by a Mosquito robot. Redundancy of both drops and temperature is productive, certainly when using 150 µl droplets, and since there is no significant difference in performance between the main screens (JCSG, LFS, HCS and HIN) these are a good set to use.

Shaw Stewart & Mueller-Dieckmann (2014 ▸) mention that 200 µl is required for HT screening in their facility, and this agrees with anecdotal experience at the SGC that expression volume is often adjusted *a priori* to ensure final yields in this volume range: smaller final volumes are awkward to handle, while larger volumes are unnecessary if they require larger volumes of expression medium. Certainly purification practice will have evolved to match the default protocols available, but it is noteworthy that the original recommendation was for only two screens (JCSG and LFS) at only one temperature, and nevertheless over the years using four screens and two temperatures has become the norm.

Although some reports advocate larger drop volumes (Chayen & Saridakis, 2008 ▸; Newman *et al.*, 2007 ▸), in our operation 150 nl has been very effective: most of our structures came directly from sparse-matrix screens set up at 150 nl, as were many optimization screens (although the exact numbers were not recorded). This corroborates reports that the dependency of crystallization on drop volume is not straightforward since the difference in surface tension may favour nucleation in nano-droplets, implying that the pros and cons of small drop volumes balance each other (Bodenstaff*et al.*, 2002 ▸; Dekker *et al.*, 2004 ▸). Furthermore, the total drop volume has not hindered successful harvesting of crystals. We therefore do not see a reason to change this drop volume, and although direct comparative data to prove it conclusively are lacking, we submit that smaller drops are effective, especially for the added exploration of concentrations, temperature and chemical space.

## 7. Recommendations for smaller volumes   

For many proteins producing 200 µl of sample is problematic, and for such cases the first priority is to know whether the sample is even likely to crystallize: in this context, the presence of any crystallinity (‘hits’, as defined before) is itself considered a ‘success’. We therefore investigated what was more informative in such situations: whether redundancy in mixing ratio or incubation temperature should be prioritized ahead of sampling more chemical conditions with different screens. Specifically, we set out to compare the following sets of strategies: (i) whether to set up more screens with fewer droplets *versus* setting up fewer screens with more droplets and (ii) whether to set up screens at multiple temperatures with fewer droplets *versus* setting up screens at single temperatures with more droplets.

We mined our data set to simulate the outcomes of both of these experiments by identifying which compromise would have resulted in the identification of fewer hits if protein volume were constant. While this allowed a rigorous analysis, it meant that the conclusions are based on proteins that are well behaved enough to produce enough sample for at least three plates: these proteins may not be representative of proteins truly limited by expression levels.

### 7.1. Multiple screens *versus* multiple droplets   

To compare whether setting up more screens yielded more hits than multiple droplets at different mixing ratios, we determined the best way of setting up 288 droplets with the same volume of protein (21.6 µl): three droplets in one screen (at 2:1, 1:1 and 1:2 mixing ratios) or three screens with one droplet each (at a 1:1 mixing ratio).

**Figure 8:**
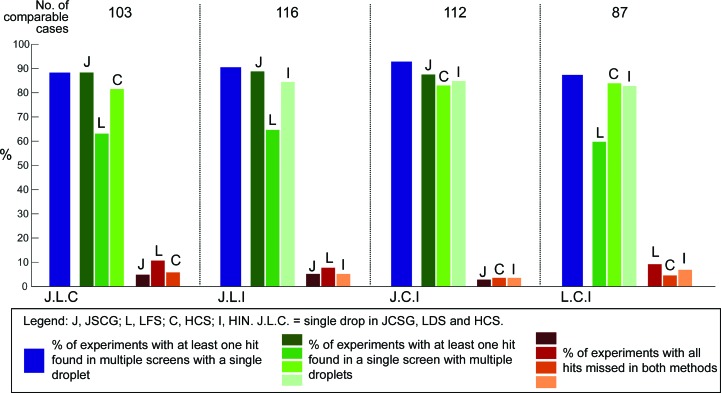
Setting up multiple screens with single droplets leads to better identification of hits when compared with setting up single screens with multiple droplets. Here, we compared the hit-identification rates for experiments with one drop at a 1:1 mixing ratio in three screens (blue bars) and with three drops at 2:1, 1:1 and 1:2 mixing ratios in a single screen (green bars) to keep the required protein sample constant. Each screen was only compared with permutations that include itself. Red bars indicate how many hits were missed by both methods.

We identified 166 groups of experiments which each consisted of three or four plates (any combinations of JCSG, LFS, HCS and HIN) containing the same protein sample (from the same purification batch and the same fresh or frozen state) at a similar concentration for crystallization, with identical compounds for co-crystallization (if any), incubated at the same temperature and set up on the same day. Each group also had at least one diffracting crystal amongst all plates in the group, to ensure that protein quality was not limiting in this analysis. The success rates for all three-screen combinations are shown in Fig. 8 ▸. For each combination, we extracted whether at least one hit was scored for (i) all three screens in 1:1 drops (dark blue bars) or (ii) any individual screen with three drops (green bars) or (iii) hits were not found in either method, therefore requiring the full three-drop/three-screen protocol (red/orange bars). While this definition of ‘success’ does rely on user scoring, as mentioned before (§[Sec sec2]2) we have found them broadly reliable even individually, without scores for later drops not being affected by hits found in earlier drops; it thus seems safe to assume that accuracy remains good when scores are effectively pooled by the criterion of at least one hit as we do here.

The comparison indicates that setting up three screens with one drop was always but only marginally more effective at finding hits than one screen with three drops (the blue bars are consistently higher than the highest green bar); in particular, the combination of JCSG, HCS and HIN was the most effective. Some of this may be attributable to the considerable overlap in conditions between JCSG and both HCS and HIN.

Of the one-screen/three-drop experiments, JCSG was consistently more effective than HIN and HCS, but only slightly so. In contrast, LFS was systematically far worse than the others when used in this context. This appears at odds with the conclusion from Fig. 6 ▸, where LFS had the highest overall success rate and also the most structures obtained directly without further optimization. The most likely explanation is that the proteins in the experiments selected here happened not to crystallize well in the narrow chemical space sampled by LFS. The corollary is that it is a screen that, when it works, it works well, often directly producing crystals sufficient for diffraction analysis; yet when it fails, it yields little information.

This leads to a further conclusion: the ‘success rate’ of screens will always depend on the proteins actually sampled during its evaluation. Thus, statements on the success rate of individual screens are meaningless unless all proteins were equally tested in all screens and followed up with equal rigour. This also applies to our analysis: while JCSG was consistently the most successful single screen, this cannot be generalized beyond this sample. The only robust conclusion is that a strategy that samples more conditions yields more hits, but only marginally so.

**Figure 9:**
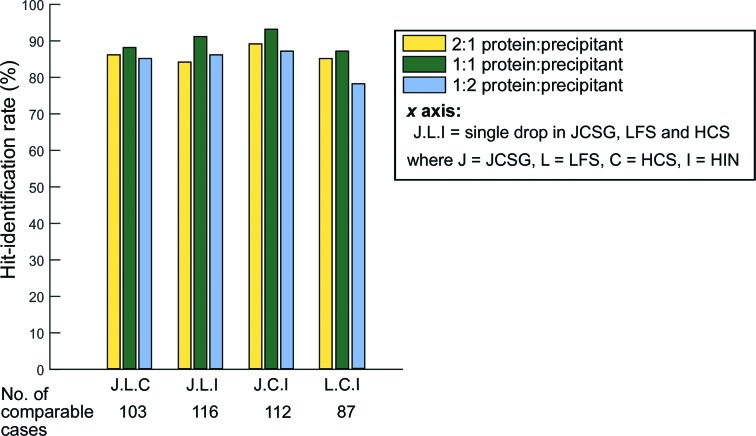
For a one-drop/multiple-screen approach, a 1:1 mixing ratio (green) consistently resulted in a better hit-identification rate compared with a 2:1 (yellow) and a 1:2 mixing ratio (blue). The screens analysed here were JCSG (J), LFS (L), HCS (C) and HIN (I); the same three-screen combinations were used as in Fig. 8 ▸.

Regarding the protein ratio in the single-drop/multi-screen approach, the 1:1 ratio had consistently higher success rates (Fig. 9 ▸). While the data set is somewhat small, with only ∼100 experiments, it is nevertheless consistent with the assumption that coarse screens were historically derived from 1:1 drop ratio crystallizations: the experiments in this subset were self-selected by the presence of diffracting crystals, which implies that the respective protein concentrations were tuned to the concentrations of the screens. In contrast, the slight bias for 2:1 drop ratios discussed in Fig. 3 ▸(*a*) is observed for the appearance of merely hits; when only plates with diffracting crystals were considered (Fig. 3 ▸ *b*) the bias becomes less apparent or disappears altogether.

### 7.2. Multiple temperatures *versus* multiple droplets   

Similarly, we compared whether setting up screens at multiple temperatures was advantageous over setting up multiple droplets at single temperatures. Once again, to keep the required protein volume constant, we chose the best way to set up a total of 192 droplets (with 28.8 µl): two droplets at one temperature (at 2:1 and 1:2 mixing ratios) or two temperatures with one droplet each (at a 1:1 mixing ratio).

We used the 587 pairs of experiments in §[Sec sec4]4 and similarly identified whether at least one hit was found if (i) two droplets at 2:1 and 1:2 mixing ratios were set up at only 277 K or only 293 K and (ii) single droplets at a 1:1 mixing ratio were set up at both these temperatures. Table 3 ▸ shows a breakdown of hits found in both methods, as well as the number of hits missed when compared with the full experiment of multiple droplets at multiple temperatures. The conclusion is similar to that of our previous analysis: sampling multiple temperatures should be prioritized over using multiple drops, since the hit-identification rate was consistently higher in the one-drop/two-temperature setup.

## 8. Protein concentration for crystallization   

**Figure 10:**
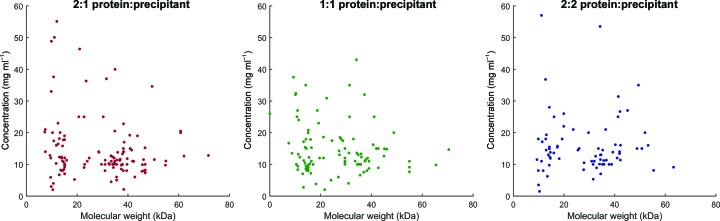
Protein sample concentration *versus* molecular weights of PDB depositions from crystals directly obtained in sparse-matrix screens at SGC Oxford, according to the protein:precipitant mixing ratio that produced the crystal responsible for the structure.

A comparison of the protein sample concentration and molecular weight for all deposited structures showed no correlation (Fig. 10 ▸) regardless of which subwell the crystals grew in. The protein sample concentrations are those measured by experimenters prior to crystallization and the molecular weight is calculated from the sequence in the database, and only structures from crystals obtained directly in the coarse screen are shown because the drop ratios for these are unambiguously linked to the subwell position. The high densities of data points at ∼13 and ∼35 kDa are from the structures of bromodomains and kinases, respectively.

It is apparent that most experimenters concentrated the proteins to ∼10 mg ml^−1^, regardless of the molecular weight of the protein; this corresponds to a widely accepted rule of thumb that is more notable for being a round number than for having ever been empirically determined. Indeed, we note a lack of correlation between molecular weight and starting concentration (∼−0.04 for all three subwells), and conclude that the value of 10 mg ml^−1^ reflects no more than an order-of-magnitude indication: each protein needs different treatment. There are no data points in the regime of the larger proteins (>60 kDa), which Wilson & DeLucas (2014 ▸) observed to require higher protein concentrations for phase separation or crystallization to occur, owing to weaker protein–protein attractive forces.

## 9. Late formation of crystals   

The duration of a crystallization experiment, *i.e.* the total time that a plate remains in the incubator, is generally determined by the likelihood of crystals appearing at any particular moment, the availability of plate storage, and hope or desperation. Regarding the former, we examined how long it took for crystals that led to PDB depositions to appear. Plates are incubated and imaged for a standard 60 days, but are available in practice for six to nine months without scheduled imaging. We estimated the latest time point for the appearance of crystals that led to deposited structures using the timestamp of the images with crystal annotation. Crystals where annotations were only found on images captured after 30 days were manually curated for accuracy; if the crystal appeared in earlier annotations but was not annotated in the earlier inspection (false positive), we corrected the time of appearance to that of the earliest image with the crystal. We also removed crystals with missing inspection data or images for visual verification.

**Figure 11:**
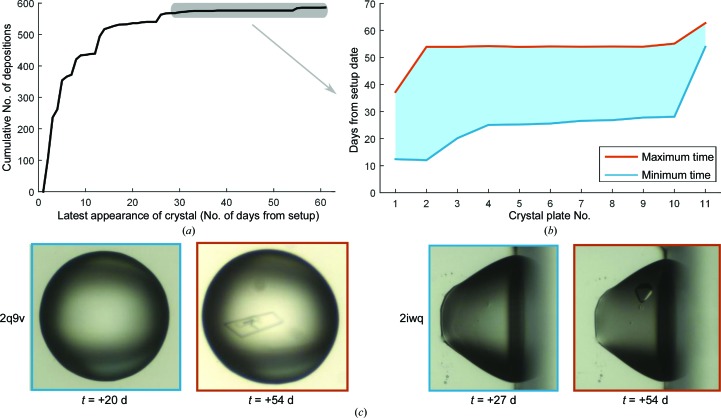
Late-appearing crystals that led to PDB depositions. (*a*) Cumulative histogram of latest estimate (in days) for the formation of these crystals. The steps on the curve correspond to typical imaging times. The earliest and latest times for crystals appearing after 30 d (grey shaded area) are shown in (*b*), where the earliest time was taken from the image before the crystals could be visually identified. (*c*) shows examples of two late-appearing crystals.

The cumulative histogram of the latest estimate of crystal appearance for the remaining 586 PDB depositions is shown in Fig. 11 ▸(*a*). Only 11 structures (2%) were from late-forming crystals, *i.e.* observed after 30 days or later. The exact times when these crystals appeared were indeterminate since automated imaging is infrequent after 7 days; nevertheless, the lower and upper bound of time can be determined using the inspection before and when the crystal was observed, as shown in Fig. 11 ▸(*b*). Since 2% of structures formed after 30 days, we conclude that it is necessary to keep plates in the incubators for 60 days.

We also found that almost 1% of hits scored by crystallo­graphers were also late-appearing crystals in an extended analysis of 15 781 plates with trackable scoring dates. Of these, 7.6% had hit annotations after 30 days of experiment setup. Since there were too many images to curate manually, the plates were filtered using *TeXRank* (Ng *et al.*, 2014 ▸), which produces a score of 0 to 1 for a droplet corresponding to its probability of containing crystals. We only visually validated 154 plates with an increase in ranking score of >0.2 from images captured before and after 30 days. Of these, 128 were verified to produce crystals, phase separation or substantial crystal growth after 30 days, whereas four targets in these plates only had annotations after 30 days, furthering our case for a 60 day incubation period.

These results are specifically derived from nanovolume sitting-drop experiments at two precise temperatures, but there are two reasons to believe that they may be generally applicable, including to larger drops or reservoirs, to hanging-drop vapour diffusion and at other temperatures. Firstly, the set of proteins are very diverse not only in size and type but also in details of preparation. Secondly, equilibration dynamics for systems in the microlitre to millilitre volume range are likely to play out on timescales of hours to days, which are shorter than the apparent time asymptote of 30–60 days. Instead, it is on the very short timescales (minutes to hours) that variation is largest, as exploited by Korczyńska *et al.* (2007 ▸) through the use of microlitre-sized reservoirs.

## 10. Conclusions   

Our analysis is based on a crystallization process that, in concert with the purification strategies at the SGC, eventually yielded diffracting crystals for 44% of all targets that had been purified. The data confirm that it is worth setting up variations of the same crystallization cocktail in order to maximize the likelihood of finding crystallization hits. Our initial strategy appears to have been effective, and the data allow us to justify a strategy for how to deploy 200 µl of protein sample in initial screens: eight plates at two temperatures (277 and 293 K) using four coarse screens (with JCSG, LFS, HCS and HIN being effective at the SGC) and three drop ratios (2:1, 1:1 and 1:2) for each condition with a drop volume of 150 nl. Where protein sample is limited, priority should be given to diversifying screening conditions and temperature rather than repeating conditions with different mixing ratios. Specifically, our data show that setting up single droplets at a 1:1 ratio with JCSG, HCS and HIN at 277 K would maximize success rates. Finally, crystals rarely appear after 60 d, so plate-storage strategies can be adjusted accordingly.

## Supplementary Material

Supporting Information.. DOI: 10.1107/S2059798315024687/nj5239sup1.pdf

## Figures and Tables

**Table 1:** Selected screens commonly used at the SGC

Screen	Abbreviation	Vendor†	Product code
JCSG+	JCSG	MD (modified)	MD1-40
Ligand Friend Screen	LFS	In-house‡	
Crystal Screen HT	HCS	HR	HR2-130
Index	HIN	HR	HR2-134
Basic ChemSpace	BCS	In-house‡	
Modern Intelligent Dynamic Alternative Screen	MIDAS	MD	MD1-60
(Emerald Bio) Precipitant Synergy Screen	EPS	JB	CS-EB-PS-B
Morpheus	MORPHEUS	MD	MD1-47
SaltRX HT	SaltRx	HR	HR2-136
MemGold	MemGold	MD	MD1-41
MemGold2	MemGold2	MD	MD1-64

† MD, Molecular Dimensions; HR, Hampton Research; JB, Jena Bioscience.

‡ In-house indicates design by SGC and formulation by MD. See Supplementary Tables S1 and S2 for the full list of conditions for our modified versions of JCSG+ and LFS.

**Table 2:** Diversity and distinct chemicals vary considerably in the four most popular screens at the SGC The internal diversity score was calculated by taking the mean of cocktail distances for all conditions, as defined by Bruno *et al.* (2014 ▸). The number of chemical components refers to the stock solutions necessary for mixing each screen.

Screen	Internal diversity score	No. of distinct chemical components
JCSG	0.5650	59
LFS	0.2728	35
HIN	0.5754	46
HCS	0.6196	54

**Table 3:** The hit-identification rate with the one-drop/two-temperature setup was consistently higher than the two-drops/one-temperature setup

	Hits in two drops/one temperature	Hits in one drop/two temperatures	No. of hits not found
277 K	479	511	17
293 K	482	511	32
