# Supplementary material for: Lessons from ten years of crystallization experiments at the SGC
Source: Acta Crystallogr D Struct Biol. 2016 Jan 22;72(Pt 2):224–35. doi: 10.1107/S2059798315024687 (PMC4756611; doi:10.1107/S2059798315024687)
Supplement: Supplementary file 1 [file d-72-00224-sup1.pdf]

STRUCTURAL BIOLOGY

Volume 72 (2016)

Supporting information for article:

Lessons from ten years of crystallization experiments at the SGC

Jia Tsing Ng, Carien Dekker, Paul Reardon and Frank von Delft

Table S1 Crystallization conditions of our modified version of JCSG+.

Row	Col	Condition
A	1	50% PEG400 -- 0.2M lithium sulfate -- 0.1M acetate pH 4.5
A	2	20% PEG3000 -- 0.1M citrate pH 5.5
A	3	20% PEG3350 -- 0.2M ammonium citrate dibasic
A	4	30% MPD -- 0.02M calcium chloride -- 0.1M acetate pH 4.5
A	5	20% PEG3350 -- 0.2M magnesium formate
A	6	20% PEG1000 -- 0.2M lithium sulfate -- 0.1M citrate pH 4.2
A	7	20% PEG8000 -- 0.1M CAPSO pH 9.5
A	8	20% PEG3350 -- 0.2M ammonium formate
A	9	20% PEG3350 -- 0.2M ammonium chloride
A	10	20% PEG3350 -- 0.2M potassium formate
A	11	50% MPD -- 0.2M ammonium phosphate dibasic -- 0.1M tris pH 8.5
A	12	20% PEG3350 -- 0.2M potassium nitrate
B	1	0.8M ammonium sulfate -- 0.1M citrate pH 4.2
B	2	20% PEG3350 -- 0.2M potassium thiocyanate
B	3	20% PEG6000 -- 0.1M bicine pH 9.0
B	4	10% PEG8000 -- 8% ethylene glycol -- 0.1M HEPES pH 7.5
B	5	40% MPD -- 5% PEG8000 -- 0.1M cacodylate pH 6.5
B	6	5% PEG1000 -- 40% ethanol -- 0.1M citrate pH 4.2
B	7	8% PEG4000 -- 0.1M acetate pH 4.5
B	8	10% PEG8000 -- 0.2M magnesium chloride -- 0.1M HEPES pH 7.0
B	9	20% PEG6000 -- 0.1M citrate pH 5.0
B	10	50% PEG200 -- 0.2M magnesium chloride -- 0.1M cacodylate pH 6.5
B	11	1.6M sodium citrate tribasic
B	12	20% PEG3350 -- 0.2M potassium citrate tribasic
C	1	20% PEG8000 -- 0.2M sodium chloride -- 0.1M citrate pH 4.2
C	2	20% PEG6000 -- 0.8M lithium chloride -- 0.1M citrate pH 4.2
C	3	20% PEG3350 -- 0.2M ammonium nitrate

C	4	10% PEG6000 -- 0.1M HEPES pH 7.0
C	5	0.8M sodium phosphate monobasic -- 0.8M potassium phosphate dibasic -- 0.1M HEPES pH 7.5
C	6	40% PEG300 -- 0.1M citrate pH 4.2
C	7	10% PEG3000 -- 0.2M zinc acetate -- 0.1M acetate pH 4.5
C	8	20% ethanol -- 0.1M tris pH 8.5
C	9	25% 1,2-propanediol -- 10% glycerol -- 0.1M sodium/potassium phosphate pH 7.5
C	10	10% PEG20000 -- 2%(v/v) dioxane -- 0.1M bicine pH 9.0
C	11	2M ammonium sulfate -- 0.1M acetate pH 4.5
C	12	10% PEG1000 -- 10% PEG8000
D	1	25% PEG1000 -- 20% glycerol
D	2	30% PEG400 -- 0.2M magnesium chloride -- 0.1M HEPES pH 7.5
D	3	50% PEG200 -- 0.2M sodium chloride -- 0.1M sodium/potassium phosphate pH 7.5
D	4	30% PEG8000 -- 0.2M lithium sulfate -- 0.1M acetate pH 4.5
D	5	60% MPD -- 0.1M HEPES pH 7.5
D	6	20% PEG8000 -- 0.2M magnesium chloride -- 0.1M tris pH 8.5
D	7	40% PEG400 -- 0.2M lithium sulfate -- 0.1M tris pH 8.5
D	8	40% MPD -- 0.1M tris pH 8.0
D	9	0.15M ammonium sulfate -- 25% PEG4000 -- 15% glycerol
D	10	40% PEG300 -- 0.2M calcium acetate -- 0.1M cacodylate pH 6.5
D	11	30% glycerol -- 15% 2-propanol -- 0.15M calcium chloride -- 0.1M acetate pH 4.5
D	12	16% PEG8000 -- 0.04M potassium phosphate dibasic -- 20% glycerol
E	1	1M sodium citrate tribasic -- 0.1M cacodylate pH 6.5
E	2	2M ammonium sulfate -- 0.2M sodium chloride -- 0.1M cacodylate pH 6.5
E	3	10% 2-propanol -- 0.2M sodium chloride -- 0.1M HEPES pH 7.5
E	4	1.26M ammonium sulfate -- 0.2M lithium sulfate -- 0.1M tris pH 8.5
E	5	40% MPD -- 0.1M CAPS pH 10.5
E	6	20% PEG3000 -- 0.2M zinc acetate -- 0.1M HEPES pH 7.5
E	7	10% 2-propanol -- 0.2M zinc acetate -- 0.1M cacodylate pH 6.5

E	8	0.8M ammonium phosphate dibasic -- 0.1M acetate pH 4.5
E	9	1.6M magnesium sulfate -- 0.1M MES pH 6.5
E	10	10% PEG6000 -- 0.1M bicine pH 9.0
E	11	16% PEG8000 -- 20% glycerol -- 0.16M calcium acetate -- 0.1M cacodylate pH 6.5
E	12	10% PEG8000 -- 0.1M tris pH 8.0
F	1	30% jeffamine M-600 -- 0.05M cesium chloride -- 0.1M MES pH 6.5
F	2	3M ammonium sulfate -- 0.1M citrate pH 5.0
F	3	20% MPD -- 0.1M tris pH 8.0
F	4	20% jeffamine M-600 -- 0.1M HEPES pH 7.5
F	5	50% ethylene glycol -- 0.2M magnesium chloride -- 0.1M tris pH 8.5
F	6	10% MPD -- 0.1M bicine pH 9.0
F	7	0.8M succinic acid
F	8	2.1M DL- malic acid
F	9	2.4M sodium malonate
F	10	1.2M sodium malonate -- 0.5% jeffamine ED-2003 -- 0.1M HEPES pH 7.0
F	11	1M succinic acid -- 1% PEG2000MME -- 0.1M HEPES pH 7.0
F	12	30% jeffamine M-600 -- 0.1M HEPES pH 7.0
G	1	30% jeffamine ED-2003 -- 0.1M HEPES pH 7.0
G	2	22% polyacrylic acid 5100 -- 0.02M magnesium chloride -- 0.1M HEPES pH 7.5
G	3	20% polyvinylpyrrolidone -- 0.01M cobalt chloride -- 0.1M tris pH 8.5
G	4	20% PEG2000MME -- 0.2M trimethylamine N-oxide -- 0.1M tris pH 8.5
G	5	12% PEG3350 -- 0.005M cobalt chloride -- 0.005M cadmium chloride -- 0.005M nickel chloride -- 0.005M magnesium chloride -- 0.1M HEPES pH 7.5
G	6	20% PEG3350 -- 0.2M sodium malonate
G	7	20% PEG3350 -- 0.1M succinic acid
G	8	20% PEG3350 -- 0.15M DL- malic acid
G	9	30% PEG2000MME -- 0.1M potassium thiocyanate
G	10	30% PEG2000MME -- 0.15M potassium bromide
G	11	2M ammonium sulfate -- 0.1M bis-tris pH 5.5

G	12	3M sodium chloride -- 0.1M bis-tris pH 5.5
H	1	0.3M magnesium formate -- 0.1M bis-tris pH 5.5
H	2	1% PEG3350 -- 1M ammonium sulfate -- 0.1M bis-tris pH 5.5
H	3	25% PEG3350 -- 0.1M bis-tris pH 5.5
H	4	45% MPD -- 0.2M calcium chloride -- 0.1M bis-tris pH 5.5
H	5	45% MPD -- 0.2M ammonium acetate -- 0.1M bis-tris pH 5.5
H	6	0.1M ammonium acetate -- 0.1M bis-tris pH 5.5 -- 16%(w/v) PEG10000
H	7	25% PEG3350 -- 0.2M ammonium sulfate -- 0.1M bis-tris pH 5.5
H	8	25% PEG3350 -- 0.2M sodium chloride -- 0.1M bis-tris pH 5.5
H	9	25% PEG3350 -- 0.2M lithium sulfate -- 0.1M bis-tris pH 5.5
H	10	25% PEG3350 -- 0.2M ammonium acetate -- 0.1M bis-tris pH 5.5
H	11	25% PEG3350 -- 0.2M magnesium chloride -- 0.1M bis-tris pH 5.5
H	12	45% MPD -- 0.2M ammonium acetate -- 0.1M HEPES pH 7.5

Table S2 Crystallization conditions of the Ligand Friendly Screen (LFS)

Row	Col	Condition
A	1	30% PEG1000 -- 0.1M SPG pH 6.0
A	2	30% PEG1000 -- 0.1M SPG pH 7.0
A	3	30% PEG1000 -- 0.1M SPG pH 8.0
A	4	60% MPD -- 0.1M SPG pH 6.0
A	5	60% MPD -- 0.1M SPG pH 7.0
A	6	60% MPD -- 0.1M SPG pH 8.0
A	7	20% PEG6000 -- 10% ethylene glycol -- 0.2M sodium chloride
A	8	20% PEG6000 -- 10% ethylene glycol -- 0.2M ammonium chloride
A	9	20% PEG6000 -- 10% ethylene glycol -- 0.2M lithium chloride
A	10	20% PEG6000 -- 10% ethylene glycol -- 0.1M magnesium chloride
A	11	20% PEG6000 -- 10% ethylene glycol -- 0.1M calcium chloride
A	12	20% PEG6000 -- 10% ethylene glycol -- 0.01M zinc chloride
B	1	30% PEG1000 -- 0.1M MIB pH 6.0
B	2	30% PEG1000 -- 0.1M MIB pH 7.0
B	3	30% PEG1000 -- 0.1M MIB pH 8.0
B	4	60% MPD -- 0.1M MIB pH 6.0
B	5	60% MPD -- 0.1M MIB pH 7.0
B	6	60% MPD -- 0.1M MIB pH 8.0
B	7	20% PEG6000 -- 10% ethylene glycol -- 0.1M MES pH 6.0 -- 0.2M sodium chloride
B	8	20% PEG6000 -- 10% ethylene glycol -- 0.1M MES pH 6.0 -- 0.2M ammonium chloride
B	9	20% PEG6000 -- 10% ethylene glycol -- 0.1M MES pH 6.0 -- 0.2M lithium chloride
B	10	20% PEG6000 -- 10% ethylene glycol -- 0.1M MES pH 6.0 -- 0.1M magnesium chloride
B	11	20% PEG6000 -- 10% ethylene glycol -- 0.1M MES pH 6.0 -- 0.1M calcium chloride
B	12	20% PEG6000 -- 10% ethylene glycol -- 0.1M MES pH 6.0 -- 0.01M zinc chloride
C	1	30% PEG1000 -- 0.1M PCB pH 6.0
C	2	30% PEG1000 -- 0.1M PCB pH 7.0
C	3	30% PEG1000 -- 0.1M PCB pH 8.0

C	4	60% MPD -- 0.1M PCB pH 6.0
C	5	60% MPD -- 0.1M PCB pH 7.0
C	6	60% MPD -- 0.1M PCB pH 8.0
C	7	20% PEG6000 -- 10% ethylene glycol -- 0.1M HEPES pH 7.0 -- 0.2M sodium chloride
C	8	20% PEG6000 -- 10% ethylene glycol -- 0.1M HEPES pH 7.0 -- 0.2M ammonium chloride
C	9	20% PEG6000 -- 10% ethylene glycol -- 0.1M HEPES pH 7.0 -- 0.2M lithium chloride
C	10	20% PEG6000 -- 10% ethylene glycol -- 0.1M HEPES pH 7.0 -- 0.1M magnesium chloride
C	11	20% PEG6000 -- 10% ethylene glycol -- 0.1M HEPES pH 7.0 -- 0.1M calcium chloride
C	12	20% PEG6000 -- 10% ethylene glycol -- 0.1M HEPES pH 7.0 -- 0.01M zinc chloride
D	1	30% PEG1000 -- 0.1M MMT pH 6.0
D	2	30% PEG1000 -- 0.1M MMT pH 7.0
D	3	30% PEG1000 -- 0.1M MMT pH 8.0
D	4	60% MPD -- 0.1M MMT pH 6.0
D	5	60% MPD -- 0.1M MMT pH 7.0
D	6	60% MPD -- 0.1M MMT pH 8.0
D	7	20% PEG6000 -- 10% ethylene glycol -- 0.1M tris pH 7.5 -- 0.2M sodium chloride
D	8	20% PEG6000 -- 10% ethylene glycol -- 0.1M tris pH 7.5 -- 0.2M ammonium chloride
D	9	20% PEG6000 -- 10% ethylene glycol -- 0.1M tris pH 7.5 -- 0.2M lithium chloride
D	10	20% PEG6000 -- 10% ethylene glycol -- 0.1M tris pH 7.5 -- 0.1M magnesium chloride
D	11	20% PEG6000 -- 10% ethylene glycol -- 0.1M tris pH 7.5 -- 0.1M calcium chloride
D	12	20% PEG6000 -- 10% ethylene glycol -- 0.1M tris pH 7.5 -- 0.01M zinc chloride
E	1	20% PEG3350 -- 10% ethylene glycol -- 0.2M sodium fluoride
E	2	20% PEG3350 -- 10% ethylene glycol -- 0.2M sodium bromide
E	3	20% PEG3350 -- 10% ethylene glycol -- 0.2M sodium iodide
E	4	20% PEG3350 -- 10% ethylene glycol -- 0.2M potassium thiocyanate
E	5	20% PEG3350 -- 10% ethylene glycol -- 0.2M sodium nitrate
E	6	20% PEG3350 -- 10% ethylene glycol -- 0.2M sodium formate
E	7	20% PEG3350 -- 10% ethylene glycol -- 0.2M sodium acetate
E	8	20% PEG3350 -- 10% ethylene glycol -- 0.2M sodium sulfate
E	9	20% PEG3350 -- 10% ethylene glycol -- 0.2M sodium/potassium tartrate

E	10	20% PEG3350 -- 10% ethylene glycol -- 0.2M sodium/potassium phosphate
E	11	20% PEG3350 -- 10% ethylene glycol -- 0.2M potassium citrate tribasic
E	12	20% PEG3350 -- 10% ethylene glycol -- 0.2M sodium malonate
F	1	20% PEG3350 -- 10% ethylene glycol -- 0.1M bis-tris-propane pH 6.5 -- 0.2M sodium fluoride
F	2	20% PEG3350 -- 10% ethylene glycol -- 0.1M bis-tris-propane pH 6.5 -- 0.2M sodium bromide
F	3	20% PEG3350 -- 10% ethylene glycol -- 0.1M bis-tris-propane pH 6.5 -- 0.2M sodium iodide
F	4	20% PEG3350 -- 10% ethylene glycol -- 0.1M bis-tris-propane pH 6.5 -- 0.2M potassium thiocyanate
F	5	20% PEG3350 -- 10% ethylene glycol -- 0.1M bis-tris-propane pH 6.5 -- 0.2M sodium nitrate
F	6	20% PEG3350 -- 10% ethylene glycol -- 0.1M bis-tris-propane pH 6.5 -- 0.2M sodium formate
F	7	20% PEG3350 -- 10% ethylene glycol -- 0.1M bis-tris-propane pH 6.5 -- 0.2M sodium acetate
F	8	20% PEG3350 -- 10% ethylene glycol -- 0.1M bis-tris-propane pH 6.5 -- 0.2M sodium sulfate
F	9	20% PEG3350 -- 10% ethylene glycol -- 0.1M bis-tris-propane pH 6.5 -- 0.2M sodium/potassium tartrate
F	10	20% PEG3350 -- 10% ethylene glycol -- 0.1M bis-tris-propane pH 6.5 -- 0.02M sodium/potassium phosphate
F	11	20% PEG3350 -- 10% ethylene glycol -- 0.1M bis-tris-propane pH 6.5 -- 0.2M potassium citrate tribasic
F	12	20% PEG3350 -- 10% ethylene glycol -- 0.1M bis-tris-propane pH 6.5 -- 0.2M sodium malonate
G	1	20% PEG3350 -- 10% ethylene glycol -- 0.1M bis-tris-propane pH 7.5 -- 0.2M sodium fluoride
G	2	20% PEG3350 -- 10% ethylene glycol -- 0.1M bis-tris-propane pH 7.5 -- 0.2M sodium bromide
G	3	20% PEG3350 -- 10% ethylene glycol -- 0.1M bis-tris-propane pH 7.5 -- 0.2M sodium iodide
G	4	20% PEG3350 -- 10% ethylene glycol -- 0.1M bis-tris-propane pH 7.5 -- 0.2M potassium thiocyanate
G	5	20% PEG3350 -- 10% ethylene glycol -- 0.1M bis-tris-propane pH 7.5 -- 0.2M sodium nitrate
G	6	20% PEG3350 -- 10% ethylene glycol -- 0.1M bis-tris-propane pH 7.5 -- 0.2M sodium formate
G	7	20% PEG3350 -- 10% ethylene glycol -- 0.1M bis-tris-propane pH 7.5 -- 0.2M sodium acetate
G	8	20% PEG3350 -- 10% ethylene glycol -- 0.1M bis-tris-propane pH 7.5 -- 0.2M sodium sulfate
G	9	20% PEG3350 -- 10% ethylene glycol -- 0.1M bis-tris-propane pH 7.5 -- 0.2M sodium/potassium tartrate

G	10	20% PEG3350 -- 10% ethylene glycol -- 0.1M bis-tris-propane pH 7.5 -- 0.02M sodium/potassium phosphate
G	11	20% PEG3350 -- 10% ethylene glycol -- 0.1M bis-tris-propane pH 7.5 -- 0.2M potassium citrate tribasic
G	12	20% PEG3350 -- 10% ethylene glycol -- 0.1M bis-tris-propane pH 7.5 -- 0.2M sodium malonate
H	1	20% PEG3350 -- 10% ethylene glycol -- 0.1M bis-tris-propane pH 8.5 -- 0.2M sodium fluoride
H	2	20% PEG3350 -- 10% ethylene glycol -- 0.1M bis-tris-propane pH 8.5 -- 0.2M sodium bromide
H	3	20% PEG3350 -- 10% ethylene glycol -- 0.1M bis-tris-propane pH 8.5 -- 0.2M sodium iodide
H	4	20% PEG3350 -- 10% ethylene glycol -- 0.1M bis-tris-propane pH 8.5 -- 0.2M potassium thiocyanate
H	5	20% PEG3350 -- 10% ethylene glycol -- 0.1M bis-tris-propane pH 8.5 -- 0.2M sodium nitrate
H	6	20% PEG3350 -- 10% ethylene glycol -- 0.1M bis-tris-propane pH 8.5 -- 0.2M sodium formate
H	7	20% PEG3350 -- 10% ethylene glycol -- 0.1M bis-tris-propane pH 8.5 -- 0.2M sodium acetate
H	8	20% PEG3350 -- 10% ethylene glycol -- 0.1M bis-tris-propane pH 8.5 -- 0.2M sodium sulfate
H	9	20% PEG3350 -- 10% ethylene glycol -- 0.1M bis-tris-propane pH 8.5 -- 0.2M sodium/potassium tartrate
H	10	20% PEG3350 -- 10% ethylene glycol -- 0.1M bis-tris-propane pH 8.5 -- 0.02M sodium/potassium phosphate
H	11	20% PEG3350 -- 10% ethylene glycol -- 0.1M bis-tris-propane pH 8.5 -- 0.2M potassium citrate tribasic
H	12	20% PEG3350 -- 10% ethylene glycol -- 0.1M bis-tris-propane pH 8.5 -- 0.2M sodium malonate
